# Modelling Cell Orientation Under Stretch: The Effect of Substrate Elasticity

**DOI:** 10.1007/s11538-023-01180-1

**Published:** 2023-07-17

**Authors:** Annachiara Colombi, Luigi Preziosi, Marco Scianna

**Affiliations:** Department of Mathematical Sciences “G.L. Lagrange”, Politecnico di Torino, Corso Duca degli Abruzzi 24, 10129 Turin, Italy

**Keywords:** Cell orientation, Mechanosensing, Cell–substrate interaction, 74B20, 74L15, 92C10, 92C37

## Abstract

When cells are seeded on a cyclically deformed substrate like silicon, they tend to reorient their major axis in two ways: either perpendicular to the main stretching direction, or forming an oblique angle with it. However, when the substrate is very soft such as a collagen gel, the oblique orientation is no longer observed, and the cells align either along the stretching direction, or perpendicularly to it. To explain this switch, we propose a simplified model of the cell, consisting of two elastic elements representing the stress fiber/focal adhesion complexes in the main and transverse directions. These elements are connected by a torsional spring that mimics the effect of crosslinking molecules among the stress fibers, which resist shear forces. Our model, consistent with experimental observations, predicts that there is a switch in the asymptotic behaviour of the orientation of the cell determined by the stiffness of the substratum, related to a change from a supercritical bifurcation scenario, whereby the oblique configuration is stable for a sufficiently large stiffness, to a subcritical bifurcation scenario at a lower stiffness. Furthermore, we investigate the effect of cell elongation and find that the region of the parameter space leading to an oblique orientation decreases as the cell becomes more elongated. This implies that elongated cells, such as fibroblasts and smooth muscle cells, are more likely to maintain an oblique orientation with respect to the main stretching direction. Conversely, rounder cells, such as those of epithelial or endothelial origin, are more likely to switch to a perpendicular or parallel orientation on soft substrates.

## Introduction and Biological Background

In the 1980s the study of cardiovascular diseases gave rise to the need of understanding the behaviour of cells undergoing periodic stretch. In fact, heart beats induce a periodic stretch not only of its own cells, but also of the arterial walls due to the induced periodic pressure and then inflation. It was then observed that smooth muscle cells in the intima of the aorta are oriented along specific directions (Buck 1979; White et al. 1983). Specifically, they either align along the vessel axis (Zhao et al. 1995) or obliquely forming helical-like structures with an angle of $20^\circ$–$40^\circ$ with respect to the vascular axial direction (Driessen et al. 2004; Gasser et al. 2006; Osborne-Pellegrin 1978). Starting from this need, Buck Buck (1980) examined the behaviour of fibroblasts seeded in vitro on a cyclically stretched rubber plastic substrate and observed that they tended to reorient more perpendicularly with respect to the stretching direction than along it. Similar experiments were then performed by many other researchers with many cells types (see Giverso et al. (submitted) for a review).

In general, it is observed that in most cell lines, such as fibroblasts, myofibroblasts, cardiomyocytes, and endothelial cells, periodic stretching triggers a dynamic response of the cytoskeleton that results in the formation of a preferred orientation of both the cytoskeleton and the cell shape itself, at either an oblique or a perpendicular angle with the main stretching direction. Actually, the aspect ratio of the cell can even reach values close to 10 with the nucleus that achieves an ellipsoidal shape with the long axis along the same direction.

In this process the leading role is played by the cytoskeleton, that is characterised by the presence of strong filamentary structures, named as stress fibres (SFs), which are bundles of aligned actin filaments cross-linked by several molecules, such as fascin, fibrin, and actinin. Then, cell focal adhesions (FAs) cluster at the ends of assembled SFs, anchoring the cell to the substrate.

Generally speaking, the action of mechanical stress induces an internal remodelling process involving the disruption, formation, and reorganization of both SFs and FAs. In this way the cell is able to adapt to the external mechanical cue, fostering changes in shape and orientation of the whole cell. Specifically, the remodelling process slowly continues untill the cell reaches a stable configuration characterised by a clearly visible mean angle between its major axis (i.e., the one defined by the main assembly of SFs) and the direction of largest stretching. It is useful to remark that cell internal orientation can occur even in the absence of effective spatial migration, i.e., it does not necessarily lead to a head-and-tail differentiated cell configuration. For this reason, we will use the word orientation, rather than polarization.

After an initial attempt to model such behaviour as a strain avoidance mechanism, based on the minimization of the sensed strain (De et al. 2008; Faust et al. 2011; Morioka et al. 2011; Safran and De 2009; Wang 2000; Wang et al. 1995), Livne et al. Livne et al. (2014) showed that a better fit was obtained by minimizing the elastic energy. This approach, firstly developed in a linear elasticity framework, was recently generalised to large deformations in Ciambella et al. (2022), Lucci and Preziosi (2021) using a very general nonlinear orthotropic elastic model.

Then, in Giverso et al. (2021) a linear viscoelastic model was proposed to explain the experimental evidence that cells reorient only if the frequency of the deformation applied to the substrate is sufficiently high, say, greater than 0.1 Hz for fibroblasts (Hsu et al. 2009; Jungbauer et al. 2008; Liu et al. 2008). In fact, this experimental fact requires the introduction of viscoelastic effects with a characteristic response time that need to be compared with the period of oscillations.

A different approach was used by Xu et al. (2016), as well as in Mao et al. (2021) and Xu et al. (2018). They schematize cells as formed by two parallel elastic struts along the orientation axis and two other struts perpendicular to them. A string connecting the ends of the struts is also considered, but then neglected. The energy of the system is then evaluated, but unfortunately it presents some flaws in the computation of the work done by the applied force. In addition, there is some confusion with some signs (see, for instance, Eqs. (13) and (18) in Xu et al. 2016). Fortunately, apart from some signs, that are a posteriori adjusted, the energy discussed is consistent with a special case of the linear elastic energy. This allows them to recover the same ordinary differential equation for the temporal evolution of cell orientation obtained by Livne et al. (2014), and thus the same equilibrium states for time independent stretches.

In the present article we use a similar approach, schematizing the cell as formed by two elastic elements that respectively model stress fiber assemblies in its main and transversal orientations (Sect. 2). The two elements are hinged at their center through a torsional elastic spring. Specifically, the elastic elements are intended to model the behaviour of both stress fibers and focal adhesions, while the torsional spring mimics the action of crosslinking molecules connecting stress fibers and resisting to shear. The mathematical model proposed is then based on the assumption that cell re-orientation results from processes occurring at different time scales, i.e., the cell readily deforms to comply to the deformation of the substrate, and actively reorients slowly remodelling its internal cytoskeletal structure to minimise its internal energy. In Sect. 3 the analytical solution of the proposed model is given and its qualitative behaviour for long times is discussed. It is shown that, in the limit of very stiff substrates, our mathematical model reduces to the models mentioned above, e.g. Livne et al. (2014), Lucci and Preziosi (2021), being then able to reproduce the related experimental results. However, the main focus of this article is to discuss the influence of the stiffness of the substrate on cell orientation. In this respect, the first fact that is observed is that the substrate need to be stiff enough to induce cell reorientation. We show that, when the stiffness of the substrate becomes much smaller than that of the stress fibers, the time to reach the equilibrium configuration increases considerably, so that reorientation is not observed in the time of the experiment, being moreover hidden by random behaviours as explained in Loy and Preziosi (in press).

The model is also able to explain the empirical observation that when the substrate is very soft, cells elongate along the main stretching direction or perpendicularly to it, rather than achieving an oblique orientation, as in the experiments reported in Terracio et al. (1988), Thodeti et al. (2009). In Sect. 4 we discuss in detail how the model is able to explain this switch in the asymptotic trend and we relate it to a change of bifurcation scenario from a supercritical one, with a stable oblique equilibrium, to a subcritical one, in which the oblique configuration becomes unstable.

Finally, in Sect. 5 we investigate the impact of cell elongation demonstrating that an increase in cell elongation leads to a larger region in the parameter space leading to either oblique or perpendicular orientations, at the expenses of the oblique orientation. This indicates that the stability switch is more likely to occur in rounder cells, such as epithelial or endothelial cells, compared to elongated cells, such as fibroblasts and smooth muscle cells.

## Mathematical Model

In order to model the cell re-orientation process described in the Introduction, it is important to observe that the phenomenon is characterised by different time scales, which allow to identify the following phases:

1. As soon as the substrate is deformed, the first reaction of the cell is to immediately adapt to the deformation of the substrate. This results in a stretching of the cell and occurs at the same time scale as the period of the imposed external deformation, which is typically of the order of one second or less.
2. Under the action of stretching, the cell then re-adjusts its cytoskeletal orientation in order to minimize the stored internal energy. This process occurs on a much longer time scale, which is in the range of several minutes. As a result of this internal remodelling process, the cell may acquire an orientation that is different from the initial one.

With this in mind, let us define a bidimensional reference frame $(\textrm{O},\textbf{i},\textbf{j})$ with the origin at the center of the mass of our representative cell and the axes aligned with the main stretching directions. If the reaction of the cell to the externally applied deformation were negligible, the deformation gradient in the plane that is applied to the substrate would be homogeneous and given by

1 $$\begin{aligned} \mathbb {F}(t) = \textrm{diag} \{1+{\varepsilon }_{xx}(t),\,1+{\varepsilon }_{yy}(t)\} = \textrm{diag}\{1 + {\varepsilon }(t),\, 1-r{\varepsilon }(t)\}, \end{aligned}$$

where *r* is a fixed constant called *biaxiality stain ratio*. In Eq. (1), the strain amplitude ${\varepsilon }(t)$ is assumed to be small and continuous in time. Actually, it was experimentally observed that strains larger than 40% can cause cell death Boccafoschi et al. (2007) and usually a strain of 10% is applied (Giverso et al. submitted). The range of *r* usually tested in experiments is $r\in [0,1]$, where $r=0$ corresponds to a substrate clamped along the sides parallel to the main stretching direction, while $r=1$ implements an isochoric planar deformation (i.e., ${\varepsilon }_{yy}=-{\varepsilon }_{xx}$). Very few results are reported for negative values of *r*, which corresponds to stretching in both directions (see, for instance, Li et al. 2016). However, the fact that the *x*-axis is assumed parallel to the maximum stretching direction implies that $r\ge -1$. At the other extremum, $r>1$ would correspond to an untested strong compression of the substrate along the *y*-axis. For this reason, we hereafter focus on the range $r\in [-1,1]$.

**Fig. 1:**
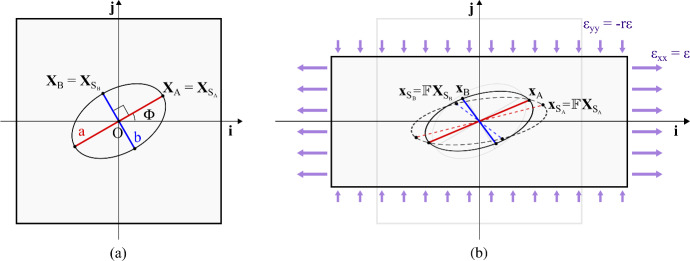
Schematization of the cell as two elastic elements that model stress fibers-focal adhesions assemblies in its main and transversal orientations. The main orientation of the cell is determined by the angle $\Phi$ (**a**) that slowly evolves due to the remodelling of the actin cytoskeleton. Due to the cell reaction to stretching, during the deformation the anchoring points ${\textbf {X}}_A$ and ${\textbf {X}}_B$ go to $\textbf{x}_A \ne \textbf{x}_{S _A }$ and $\textbf{x}_B \ne \textbf{x}_{S _B }$ (**b**), where, for instance, $\textbf{x}_{S _A }$ is the location of the point that would be occupied by ${\textbf {X}}_A$ as a result of the imposed deformation in absence of cell reaction (Color figure online)

As shown in Fig. 1, the cell cytoskeleton is schematised by two elastic elements, named *a* and *b*, with elastic moduli $$k_a $$ and $$k_b $$ and end points $$A ,A '$$ and $$B ,B '$$, respectively. They are in charge of describing the response of the cell to the external mechanical input: the element *a* along the main orientation of SFs, hereafter characterised through the time-dependent angle $$\Phi $$, and the element *b* along a transversal (i.e., rotated of an angle $$\theta $$) direction. These two elements are further assumed to be:

- linked to the substrate at their end points, which indeed model focal adhesion complexes. Specifically, we denote as $S _{Q }$ the point of the substrate that is connected with the end point *Q* of one of the two cytoskeletal elements of the cell (being $Q \in \{A ,A ',B ,B '\}$);
- hinged at their centers (which are superimposed to the domain origin $$O $$);
- interconnected by a torsional spring, with modulus equal to $k_{\theta }$, that is at rest when the two elements are orthogonal, i.e., when $\theta =\frac{\pi }{2}$;
- the two cytoskeletal elements have relaxed lengths equal to $$2L_a $$ and $$2L_b $$, respectively, which can be interpreted as mean dimensions of the cell in the absence of stresses. They are assumed to be constant.

Let us now model the above-introduced first phase of the process, i.e., the cell instantaneous response to the imposed substrate deformation. To do this, we hereafter take advantage of the symmetry of the system and focus on the upper half of the domain.

Before stretching, the cell is in a rest configuration. This, as seen, implies orthogonal elastic elements with semi-lengths $$L_a $$ and $$L_b $$, respectively, with main SF orientation defined by the angle $$\Phi $$. The two end points of interest occupy domain locations $${\textbf {X}}_A $$ and $${\textbf {X}}_B $$ that coincide with the locations of the substrate points to which they are linked to, i.e., $${\textbf {X}}_A = {\textbf {X}}_{S _A }$$ and $${\textbf {X}}_B = {\textbf {X}}_{S _B }$$ (see Fig. 1a).

As a consequence of the imposed deformation, in absence of cell reaction, the two substrate points of our interest would go to $$\textbf{x}_{S _A }=\mathbb {F}{\textbf {X}}_{S _A }$$ and $$\textbf{x}_{S _B }=\mathbb {F}{\textbf {X}}_{S _B }$$ (see Fig. 1b). However, the cell is not passively dragged by such a substrate displacement, but it rather resists to the underlying stretch. So, the end points $$A $$ and $$B $$ shift to $$\textbf{x}_A \ne \textbf{x}_{S _A }$$ and $$\textbf{x}_B \ne \textbf{x}_{S _B }$$. In this respect, the points $$\textbf{x}_A $$ and $$\textbf{x}_B $$ are identified by the angles $$(\Phi -\theta _a )$$ and $$(\Phi +\pi /2+\theta _b )$$ and by the distances from the origin $$L_a =L_a + \xi _a $$ and $$L_b =L_b +\xi _b $$, respectively. In particular, $$\theta _a $$ and $$\theta _b $$ give the variation of the relative orientation of the two cytoskeletal elastic elements, whereas $$\xi _a $$ and $$\xi _b $$ give the variation of their lengths. The effective values of these quantities can be obtained by the minimization of the *total energy* stored by the system as a consequence of the imposed substrate deformation

2 $$\begin{aligned} U_{\text {tot}}=\dfrac{1}{2}k_a \xi _a ^2+\dfrac{1}{2}k_b \xi _b ^2 +\dfrac{1}{2}k_\theta (\theta _a +\theta _b )^2 +\dfrac{1}{2}k_s \left( |\textbf{x}_{S _A }-\textbf{x}_A |^2+|\textbf{x}_{S _B }-\textbf{x}_B |^2\right) ,\nonumber \\ \end{aligned}$$

where the last term is an elastic contribution that accounts for the above-explained mechanical resistance of the cell to the dragging due to the substrate deformation, being $$k_s $$ the corresponding elastic modulus. As already mentioned, the Hooke constants of the elements along *a* and *b* are respectively $$k_a $$ and $$k_b $$, while $$k_\theta $$ is the torsion coefficient of the torsional spring in $$O $$. In the limit of small deformations, i.e., for $$\theta _a , \theta _b , \dfrac{\xi _a }{L_a }, \dfrac{\xi _b }{L_b } \ll 1$$, $$U_{\text {tot}}$$ simplifies to

3 $$\begin{aligned} U_{\text {tot}}= & {} \dfrac{1}{2}k_a \xi _a ^2+\dfrac{1}{2}k_b \xi _b ^2+\dfrac{1}{2}k_\theta (\theta _a +\theta _b )^2 \nonumber \\{} & {} + \dfrac{1}{2}k_s \big [{\varepsilon }^2 L_a ^2(\cos ^2\Phi +r^2\sin ^2\Phi ) -\, 2{\varepsilon }L_a ^2(1+r)\sin \Phi \cos \Phi \,\theta _a + L^2_a \theta _a ^2\nonumber \\{} & {} - 2{\varepsilon }L_a \xi _a (\cos ^2\Phi -r\sin ^2\Phi ) + \xi _a ^2 \big ] \nonumber \\{} & {} +\,\dfrac{1}{2}k_s \big [{\varepsilon }^2 L_b ^2(\sin ^2\Phi +r^2\cos ^2\Phi ) - 2{\varepsilon }L_b ^2(1+r)\sin \Phi \cos \Phi \,\theta _b +L^2_b \theta _b ^2 \nonumber \\{} & {} +\,2{\varepsilon }L_b \xi _b (r\cos ^2\Phi -\sin ^2\Phi ) +\xi _b ^2\big ], \end{aligned}$$

(see the “Appendix” for more details on the technical calculations), which is minimised by a cell configuration characterised by

4 $$\begin{aligned} \left\{ \begin{array}{l} \bar{\xi }_a =\dfrac{{\varepsilon }L_a k_s }{k_a +k_s } (\cos ^2\Phi -r\sin ^2\Phi ),\\ \bar{\xi }_b =-\,\dfrac{{\varepsilon }L_a k_s }{k_b +k_s } (r\cos ^2\Phi -\sin ^2\Phi ),\\ \bar{\theta }_ab :=\bar{\theta }_a + \bar{\theta }_b =\dfrac{2{\varepsilon }k_s }{\left( \frac{1}{L_a ^2}+\frac{1}{L_b ^2}\right) k_\theta +k_s }(1+r)\sin \Phi \cos \Phi . \end{array}\right. \end{aligned}$$

So, for a given $\Phi$, under the externally imposed stretch, we assume that the cell readily achieves a cytoskeletal arrangement defined, in terms of SF lengths and orientations, by the equilibrium quantities in Eq. (4). In this respect, being an equilibrium, the viscosity of the cytoskeleton does not play any role. The influence of the cell membrane is also neglected. However, if it is schematised as an elastic spring connecting the end points of the two elements it would lead to a contribution that can be absorbed in the coefficients of Eq. (4).

Let us now focus on the slower remodeling process. In order to do that, we assume that the temporal evolution of $\Phi$ is driven by the virtual work of the forces *exerted on the cell cytoskeleton* due to the externally imposed deformation of the substrate. In the elastic case such a virtual work can be written in terms of the derivative of the internal potential energy

$$\begin{aligned} U_{\text {in}}=\dfrac{1}{2}k_a \bar{\xi }_a ^2+\dfrac{1}{2}k_b \bar{\xi }_b ^2+\dfrac{1}{2}k_\theta \bar{\theta }^2_{ab }. \end{aligned}$$

In addition, there is a dissipation force that resists to remodelling and eventually results in the application of a viscous-like moment proportional to the angular velocity. So, neglecting inertia, the evolution of cytoskeletal reorientation can be described by the following equation

5 $$\begin{aligned} 0=-h\,\dfrac{d\Phi }{dt} -\dfrac{\partial U_{\text {in}}}{\partial \Phi }, \end{aligned}$$

where *h* is related to the characteristic time for stress fiber and focal adhesion renewal dynamics.

Recalling Eq. (4), we have that

6 $$\begin{aligned} U_{\text {in}}= & {} \dfrac{1}{2}{\varepsilon }^2 k_s ^2 \left\{ \dfrac{k_a L_a ^2}{(k_a +k_s )^2}(\cos ^2\Phi -r\sin ^2\Phi )^2 + \dfrac{k_b L_b ^2}{(k_b +k_s )^2}(r\cos ^2\Phi -\sin ^2\Phi )^2\right. \nonumber \\{} & {} \left. +\,\dfrac{4k_\theta }{\left[ \left( \frac{1}{L_a ^2}+\frac{1}{L_b ^2}\right) k_\theta +k_s \right] ^2} (1+r)^2\sin ^2\Phi \cos ^2\Phi \right\} , \end{aligned}$$

and Eq.(5) therefore rewrites as

7 $$\begin{aligned} h\,\dfrac{d\Phi }{dt}= & {} 2{\varepsilon }^2(t) k_s ^2 (1+r) \left\{ \dfrac{k_a L_a ^2}{(k_a +k_s )^2}[(1+r)\cos ^2\Phi -r] \right. \nonumber \\{} & {} \left. +\dfrac{k_b L_b ^2}{(k_b +k_s )^2}[(1+r)\cos ^2\Phi -1] \right. \nonumber \\{} & {} \left. -\,\dfrac{2k_\theta }{\left[ \left( \frac{1}{L_a ^2}+\frac{1}{L_b ^2}\right) k_\theta +k_s \right] ^2} (1+r)[2\cos ^2\Phi -1]\right\} \sin \Phi \cos \Phi . \end{aligned}$$

We firstly notice that the case $r=-1$, corresponding to the equi-biaxial deformation with ${\varepsilon }_{yy}={\varepsilon }_{xx}$, will lead to no evolution of $\Phi$. In fact, from the mathematical viewpoint the r.h.s. of Eq. (9) vanishes and from the mechanical viewpoint it corresponds to an isotropic deformation with equal strain in all directions.

We also observe that in the limit $k_s \gg k_a ,k_b , \dfrac{k_\theta }{L^2}$ (where $\frac{1}{L^2}=\frac{1}{L_a ^2}+\frac{1}{L_b ^2}$), Eq. (7) is identical to the evolution equation considered in Livne et al. (2014) for the case $k_\theta =0$, and it can be also obtained from the model proposed in Giverso et al. (2021) by neglecting viscoelastic effects and second order mixed energy terms in the quadratic form of the elastic energy.

In addition, softening the substrate leads to a decrease of the forcing term in Eq. (7). Therefore, the dynamics slow down with the characteristic time that increases like $$k_s ^{-2}$$ as $$k_s $$ decreases.

Lastly, we remark that we are only focusing on orientational changes and not on shape change, that is, the rest length of the axes of the cells do not remodel.

For the following discussion, it is useful to scale the mechanical coefficients in Eq. (7) with $$k_a $$ and introduce the dimensionless quantities

8 $$\begin{aligned} \hat{k}_b =\dfrac{k_b }{k_a }, \qquad \hat{k}_s =\dfrac{k_s }{k_a }, \qquad \hat{k}_\theta =\dfrac{2k_\theta }{k_a L_a ^2}, \qquad \end{aligned}$$

and the aspect ratio $\lambda ={L_b ^2}/{L_a ^2}$.

Then, by scaling times with $\tau =\dfrac{h}{k_a L_a ^2}$, we can rewrite Eq. (7) as

9 $$\begin{aligned} \begin{aligned} \dfrac{d\Phi }{d\hat{t}} =&\,\dfrac{2\hat{k}_s ^2}{(1+\hat{k}_s )^2}\,{\varepsilon }^2(t)\,(1+r)^2(\alpha \cos ^2\Phi -\beta ) \sin \Phi \cos \Phi , \end{aligned} \end{aligned}$$

where

10 $$\begin{aligned} \alpha= & {} 1+\lambda \hat{k}_b \left( \dfrac{1+\hat{k}_s }{\hat{k}_b +\hat{k}_s }\right) ^2- 2\hat{k}_\theta \left( \dfrac{ 1+\hat{k}_s }{\frac{\lambda +1}{2\lambda }\hat{k}_\theta +\hat{k}_s }\right) ^2,\nonumber \\ \beta= & {} \dfrac{r}{1+r}+\dfrac{\lambda \hat{k}_b }{1+r}\left( \dfrac{1+\hat{k}_s }{\hat{k}_b +\hat{k}_s }\right) ^2- \hat{k}_\theta \left( \dfrac{ 1+\hat{k}_s }{\frac{\lambda +1}{2\lambda }\hat{k}_\theta +\hat{k}_s }\right) ^2 . \end{aligned}$$

Interestingly, $\alpha$ is independent from the biaxiality ratio *r*, while the mechanical parameters, i.e., $\hat{k}_b$ $\hat{k}_\theta$, $\hat{k}_s$, and the aspect ratio $\lambda$ influence both $\alpha$ and $\beta$.

## Analytic Solution and Asymptotic Behaviour

The analytical solution of Eq. (9) can be written in an implicit form as

11 $$\begin{aligned} \dfrac{|\alpha \cos ^2\Phi (\hat{t})-\beta |^{\alpha /(\beta \gamma )}}{[\cos ^2\Phi (\hat{t})]^{1/\beta }[\sin ^2\Phi (\hat{t})]^{1/\gamma }}= C\exp \left[ -\dfrac{4\hat{k}_s ^2}{(1+\hat{k}_s )^2}(1+r)^2\int _0^{\hat{t}}{\varepsilon }^2(\hat{\tau })\,d\hat{\tau }\right] , \end{aligned}$$

where *C* is the integration constant depending on the initial value $\Phi (0)$ and

12 $$\begin{aligned} \gamma =\alpha -\beta =\dfrac{1}{1+r}+\dfrac{r}{1+r}\lambda \hat{k}_b \left( \dfrac{1+\hat{k}_s }{\hat{k}_b +\hat{k}_s }\right) ^2- \hat{k}_\theta \left( \dfrac{ 1+\hat{k}_s }{\frac{\lambda +1}{2\lambda }\hat{k}_\theta +\hat{k}_s }\right) ^2. \end{aligned}$$

Though it is not immediate to visualize the behaviour of the solution, it can be noticed that, for any ${\varepsilon }(\hat{t})$, for large times the r.h.s. of Eq. (11) will eventually vanish. So, the asymptotic behaviour of $\Phi (t)$ is governed by the sign of the exponents in the factors of the l.h.s. of Eq. (11), i.e. it is substantially dictated by the sign of $\alpha$, $\beta$, and $\gamma$. In fact, by recalling that $|{\varepsilon }_{xx}|>|{\varepsilon }_{yy}|$ implies $r>-1$ and defining $\Phi _{obl }^{eq }$ such that

13 $$\begin{aligned} \cos ^2\Phi _{obl }^{eq }=\frac{\beta }{\alpha } , \end{aligned}$$

one has the following Proposition

### Proposition

For $$r>-1$$

- If $$\alpha>\beta >0$$, then $$\Phi (t)\rightarrow \Phi _{obl }^{eq }$$;
- If $\alpha <\beta$ and $\beta >0$, then $\Phi (t)\rightarrow \Phi _\Vert ^{eq }=0$;
- If $\alpha<\beta <0$, then either $\Phi (t)\rightarrow \Phi _\Vert ^{eq }=0$, or $\Phi (t)\rightarrow \Phi _\perp ^{eq }=\frac{\pi }{2}$;
- If $\alpha >\beta$ and $\beta <0$, then $\Phi (t)\rightarrow \Phi _\perp ^{eq }=\frac{\pi }{2}$.

**Fig. 2:**
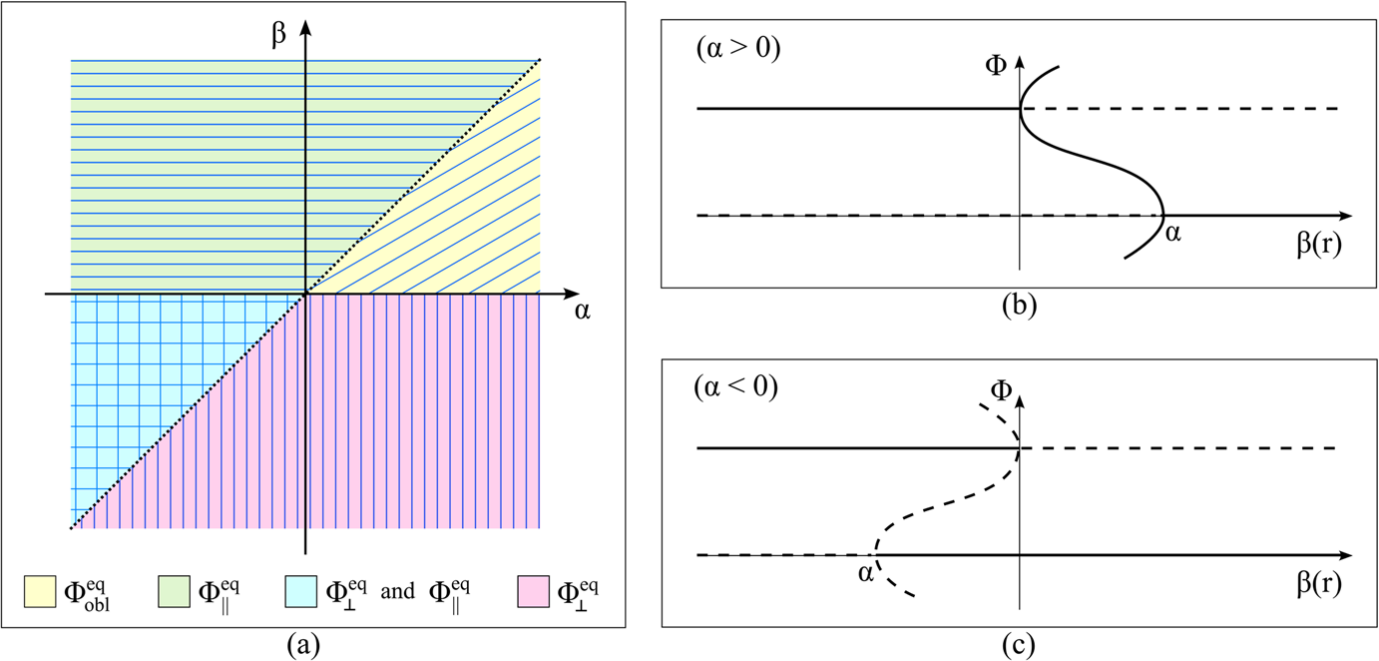
**a** Summary of the asymptotic trends in the $(\alpha , \beta )$ plane. In the panels on the right, the bifurcation diagrams for a time indepedent strain are reported for **b** $\alpha >0$ and **c** $\alpha <0$. In the former case the oblique orientation is stable when it exists and bifurcations are supercritical. In the latter it is unstable and bifurcations are subcritical. In particular, only the parallel and/or perpendicular orientations are stable (Color figure online)

### Proof

Let us start observing that $\Phi (t)=\frac{\pi }{2}$ can nullify the l.h.s. of Eq. (11) only if $\beta <0$ (pale blue and magenta regions in Fig. 2a). $\square$

Similarly, $\Phi (t)=0$ can do it only if $\gamma <0$, that is, if $\alpha <\beta$ (pale blue and green regions in Fig. 2a).

Regarding the oblique orientation $\Phi _{obl }^{eq }$, Eq. (13) implies that it exists only if $0\le \dfrac{\beta }{\alpha }\le 1$ (i.e., the pale blue and yellow regions in Fig. 2a). So, the fact that the ratio ${\beta }/{\alpha }$ must be positive implies that the l.h.s. of Eq. (11) can be nullified when $\Phi =\Phi _{obl }^{eq }$ only for $\gamma >0$, i.e. $\alpha >\beta$. This rules out the possibility of negative values of $\alpha$ and $\beta$, and leaves only the possibility $\alpha>\beta >0$, that is the yellow region in Fig. 2a.

We observe that Eq. (13) is identical to the oblique orientation found in Giverso et al. (2021), Livne et al. (2014), Lucci and Preziosi (2021), where, however, the effect of substratum deformability was not taken into account. We also notice that the asymptotic values just identified remain the same if one looks at the limit case of a time independent strain. This allows to discuss the possible scenarios using classical stability tools and terminologies, as done in previous papers (see, for instance, Giverso et al. 2021; Lucci and Preziosi 2021).

Before proceeding with it, we highlight that, taking separately the three terms on the r.h.s. of Eq. (7), for a time independent stretch, the first two terms tend to destabilize the equilibrium orientations $\Phi _\Vert ^{eq }$ and $\Phi _\perp ^{eq }$, while the last one tends to stabilize them. The opposite holds true for $\Phi _{obl }^{eq }$. As we will see in Sect. 4, this will lead to important switches in the asymptotic behaviour also seen in some experiments (see, for instance, Terracio et al. 1988; Thodeti et al. 2009). Specifically, referring to Fig. 2b, we can observe that for $\alpha >0$

- $\Phi _\Vert ^{eq }$ is stable for $\beta \ge \alpha$;
- $\Phi _\perp ^{eq }$ is stable for $\beta \le 0$;
- $\Phi _{obl }^{eq }$ is stable whenever it exists, i.e. when $0\le \beta \le \alpha$.

On the other hand, referring to Fig. 2c, for $\alpha <0$

- $\Phi _\Vert ^{eq }$ is stable for $\beta \ge \alpha$;
- $\Phi _\perp ^{eq }$ is stable for $\beta <0$;
- $\Phi _{obl }^{eq }$ is always unstable.

Therefore, in the case of a time independent strain the system is characterised by a switch from a supercritical to a subcritical bifurcation scenario when $\alpha$ changes sign from positive to negative. In the following, we will thus extend this classification, calling supercritical (resp. subcritical) scenario the one with $\alpha >0$ (resp. $\alpha <0$).

## Effect of Substratum Stiffness

As $\alpha$ and $\beta$ both depend on $\hat{k}_s$, the effect of the elasticity of the substratum is not immediately clear, because it influences both dimensionless parameters at the same time. On the other hand, the aim of the article is to highlight the effect of the substratum on the evolution of cell orientation. Therefore, hereafter we will translate the discussion done in the previous section in terms of substrate stiffness to prove that it can determine a switch in the asymptotic behaviour. In particular, we want to show that for decreasing values of the substrate stiffness, the bifurcation landscape can change from a supercritical one as in Giverso et al. (2021), Lucci and Preziosi (2021) to a subcritical one with an unstable oblique orientation and stable parallel and/or perpendicular orientations, as observed in Terracio et al. (1988), Thodeti et al. (2009).

Before doing that we make some comments on the ammissible physiological range of the parameters, so that we can limit the discussion to the biologically relevant dynamics. First of all, from phenomenological observations, we can certainly restrict our attention to $$\lambda \in (0,1]$$ (i.e., $$0<L_b \le L_a $$) because experiments show that cells are more elongated along the main direction, i.e., along the element *a* rather than along *b*. In addition, from the mechanical point of view, it can also be observed that since the response of cells to deformations along the main orientation is stronger than along the perpendicular one (even much stronger), we can take $$\hat{k}_b \in (0,1)$$ (i.e., $$0<k_b <k_a $$). Similarly, we expect $$\hat{k}_\theta \in \left( 0,\frac{1}{2}\right) $$. This can be realised comparing, for the same deformation involving the opening of the angle in *O* for a round cell, the energetic terms related to the torsional spring (proportional to $$k_\theta $$) with the energy that would be stored by a spring with rigidity $$k_a $$ connecting the extrema of the elements *a* and *b*.

So, having in mind the above biologically sound constraints, we will focus the following discussion on the subspace of parameters defined by

14 $$\begin{aligned} r\in (-1,1],\quad \lambda \in (0,1], \quad \hat{k}_b \in (0,1), \quad \textrm{and}\quad \hat{k}_\theta \in \left( 0,\frac{1}{2}\right) , \end{aligned}$$

though it could be easily extended outside this range.

**Fig. 3:**
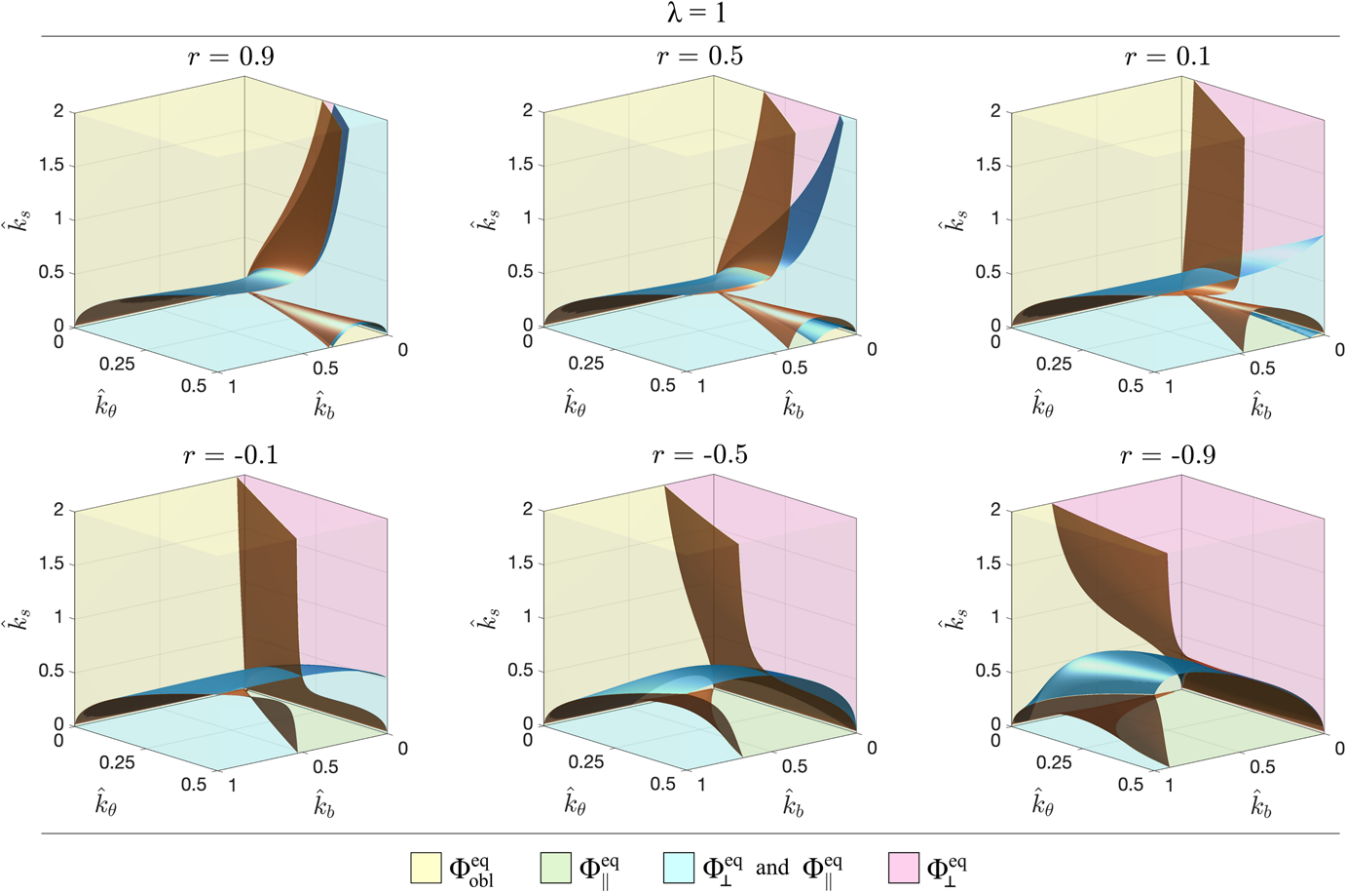
Surfaces $\gamma (\hat{k}_b , \hat{k}_\theta ,\hat{k}_s ,r)=0$ in blue, and to $\beta (\hat{k}_b , \hat{k}_\theta ,\hat{k}_s ,r)=0$ in brown that delimit the different scenarios in the case of round cells, i.e., with $\lambda =1$, for different values of *r*, positive in the top row, negative below

In Fig. 3 and then in Fig. 6 we plot, respectively for round cells $\lambda =1$ and elongated cells with $\lambda =0.5$, the surfaces corresponding to $\gamma (\hat{k}_b , \hat{k}_\theta ,\hat{k}_s ,r)=0$ in blue and to $\beta (\hat{k}_b , \hat{k}_\theta ,\hat{k}_s ,r)=0$ in brown for several fixed values of *r*. These surfaces subdivide the space of parameters in regions characterised by the different asymptotic behaviours, that are identifiable by the same colours as in Fig. 2a.

Focusing on the coordinate planes, we have that in the limit $\hat{k}_\theta =0$, if $r>0$ then $\beta$ and $\gamma$ are both positive, and so, the solution can only tend to $\Phi _{obl }^{eq }$. If, instead, $r<0$, that corresponds to imposing a tension in both directions, then the solution will tend toward $\Phi _{obl }^{eq }$ when

$$\begin{aligned} \hat{k}_s \in \left[ \dfrac{\sqrt{-r\lambda \hat{k}_b }-\hat{k}_b }{1-\sqrt{-r\lambda \hat{k}_b }}, \dfrac{\sqrt{\hat{k}_b }-\sqrt{\frac{-r}{\lambda }}\hat{k}_b }{\sqrt{\frac{-r}{\lambda }}-\sqrt{\hat{k}_b }} \right] , \end{aligned}$$

to $\Phi _\perp ^{eq }$ above this interval and to $\Phi _\Vert ^{eq }$ below it.

On the other hand, in the limit $\hat{k}_b =0$, if $r<0$, then $\beta$ is always negative. So, $\Phi _\perp ^{eq }$ is always attractive and $\Phi _\Vert ^{eq }$ might be for softer values of $\hat{k}_s$ below the blue surface $\gamma =0$, i.e, for

15 $$\begin{aligned} \hat{k}_s <k_s ^\textrm{low}:=\dfrac{\sqrt{r+1}-\Lambda \sqrt{\hat{k}_\theta }}{1-\sqrt{(r+1)\hat{k}_\theta }}\sqrt{\hat{k}_\theta }, \end{aligned}$$

where $\Lambda =\dfrac{\lambda +1}{2\lambda }$, if $k_s ^\textrm{low}$ is positive. We explicitly remark that in this case a trend toward $\Phi _{obl }^{eq }$ is ruled out. In addition, if $r<-1+\dfrac{\Lambda ^2}{2}$, then $k_s ^\textrm{low}$ becomes negative for $\hat{k}_\theta >\dfrac{1+r}{\Lambda ^2}$. In this case also the trend toward $\Phi _\Vert ^{eq }$ is ruled out and the solution can only tend to $\Phi _\perp ^{eq }$.

Instead, in order to describe what happens in the limit $\hat{k}_b =0$ when $r>0$, corresponding to the usually tested cases, we start noticing that since $\gamma =\beta +\dfrac{1-r}{1+r}>\beta$ (we recall that $r<1$), the brown surface $\beta =0$ is always above the blue one $\gamma =0$. Hence, the solution will tend toward $\Phi _{obl }^{eq }$ above the brown surface, i.e., for

16 $$\begin{aligned} \hat{k}_s >k_s ^\textrm{up}:=\dfrac{\Lambda \sqrt{r\hat{k}_\theta }-\sqrt{r+1}}{\sqrt{(r+1)\hat{k}_\theta }-\sqrt{r}}\sqrt{\hat{k}_\theta }, \end{aligned}$$

and to $\Phi _\perp ^{eq }$ for values of $\hat{k}_s$ below $k_s ^\textrm{up}$, with $\Phi _\Vert ^{eq }$ that becomes also attractive for suitable initial conditions when the condition (15) is satisfied.

**Fig. 4:**
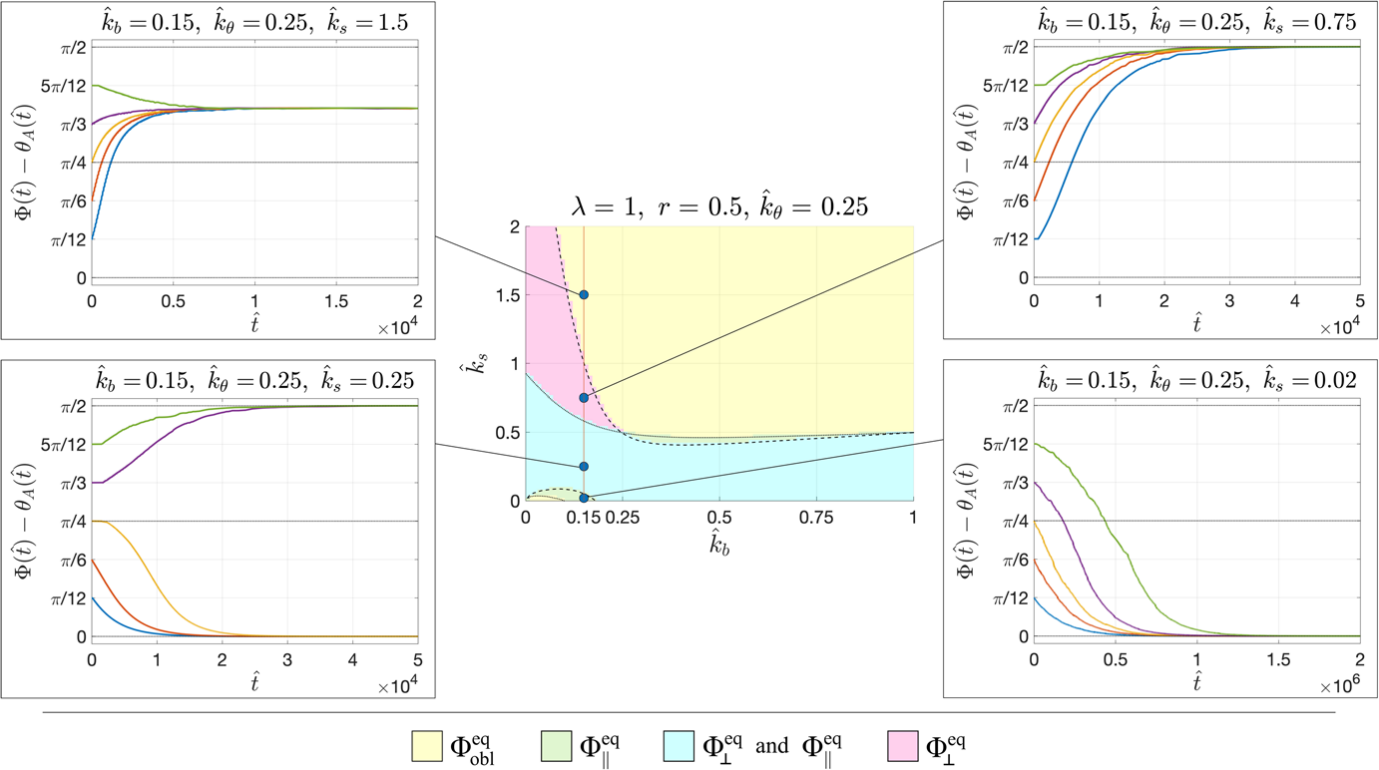
Examples of evolutions of the orientations in response of an imposed strain ${\varepsilon }(\hat{t})=0.05\left( 1-\cos \frac{\hat{t}}{10}\right)$; toward $\Phi _{obl }^{eq }$ in the yellow region (top left); $\Phi _\perp ^{eq }$ in the magenta region (top right); either $\Phi _\Vert ^{eq }$ or $\Phi _\perp ^{eq }$ according to the initial condition in the cyan region (bottom left); only $\Phi _\Vert ^{eq }$ in the green region (bottom right). The dotted line corresponds to $\beta =0$ and the full line to $\gamma =0$

In order to describe what happens for $\hat{k}_b ,\hat{k}_\theta \ne 0$, for decreasing values of $\hat{k}_s$, we use as a representative example the case with $\lambda =1$ and $r=0.5$, corresponding to an isocoric deformation, and we fix $\hat{k}_\theta =0.25$ (see Fig. 4).

According to where the parameter values fall, one has a trend toward $\Phi _{obl }^{eq }$ (yellow region), $\Phi _\perp ^{eq }$ (magenta region), $\Phi _\Vert ^{eq }$ (green region) or either $\Phi _\perp ^{eq }$ or $\Phi _\Vert ^{eq }$ (cyan region). In particular, fixing $\hat{k}_b =0.5$ one has that for stiffer substrates (namely, $\hat{k}_s >0.46$) the solution will tend toward $\Phi _{obl }^{eq }$. For softer substrates with $0.41<\hat{k}_s <0.46$ the solution will tend toward $\Phi _\Vert ^{eq }$ and for even softer substrates with $\hat{k}_s <0.41$ the solution may also tend toward $\Phi _\perp ^{eq }$ according to the initial orientation.

For very low values of $\hat{k}_b$ (e.g., $\hat{k}_b =0.15$) one has that for a very high stiffness, i.e., $\hat{k}_s >1$, again the solution will tend toward $\Phi _{obl }^{eq }$. For softer substrates (i.e., $0.05<\hat{k}_s <1$) the solution will tend toward $\Phi _\perp ^{eq }$, with the parallel orientation that adds up when $0.05<\hat{k}_s <0.58$. Then, for very soft substrates (i.e., $\hat{k}_s <0.05$) the solution can only tend toward $\Phi _\Vert ^{eq }$.

**Fig. 5:**
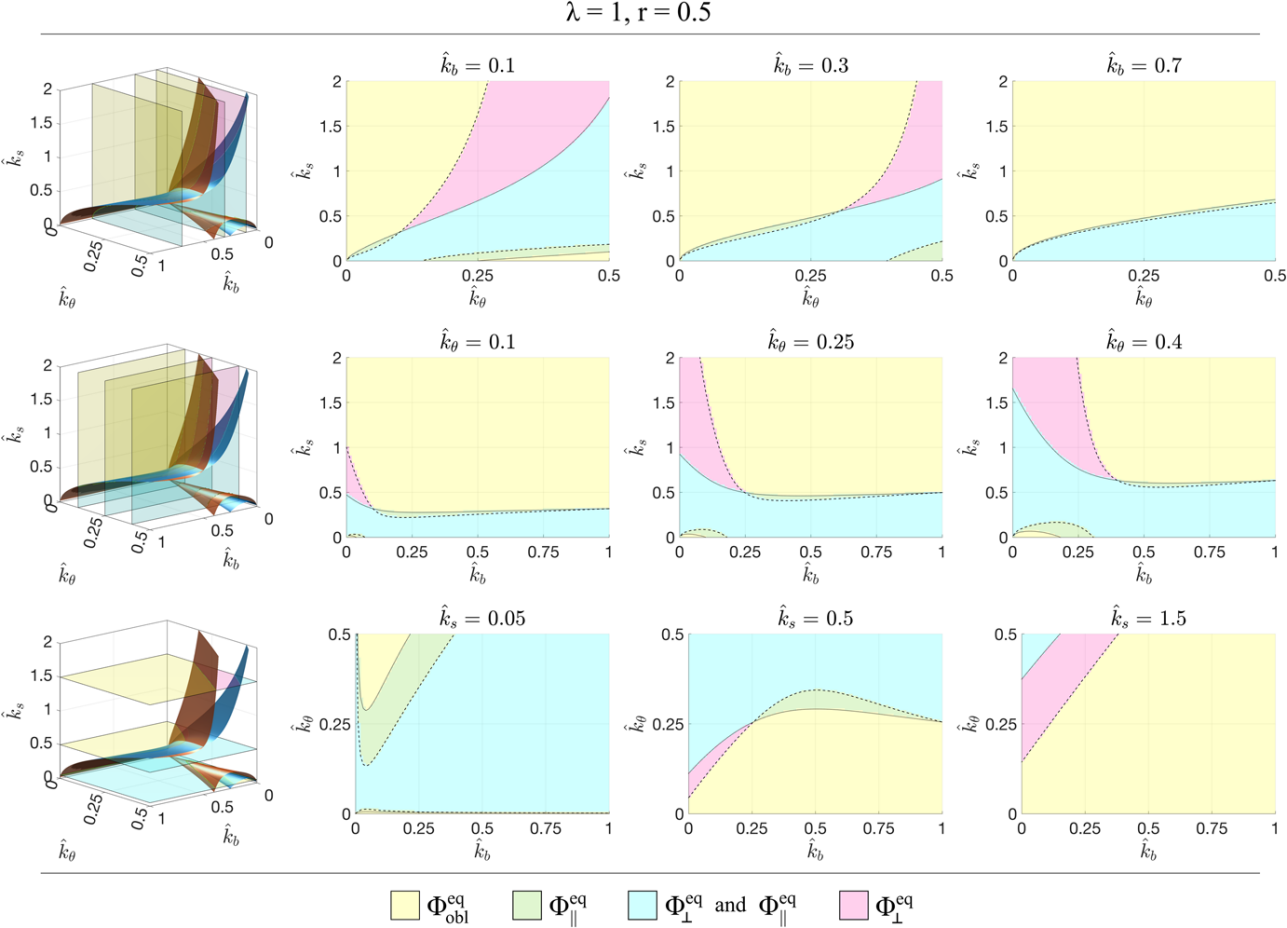
Sections for constant $\hat{k}_b$ (top row), constant $\hat{k}_\theta$ (central row), and constant $\hat{k}_s$ (bottom row) for $\lambda =1$ and $r=0.5$. The dotted line corresponds to $\beta =0$ and the full line to $\gamma =0$

From the central row of Fig. 5, it can be observed that variations in the value of $\hat{k}_\theta =0.25$ (that is the ratio between cell resistence to shear and the cell elastic modulus in its main direction) lead to similar trends, obviously with different values of the parameters leading to transitions. In particular, generally speaking, lower values of $\hat{k}_\theta$ lead to larger regions characterised by a trend toward $\Phi _{obl }^{eq }$ and smaller regions characterised by a trend toward $\Phi _\Vert ^{eq }$ and/or $\Phi _\perp ^{eq }$. This means that resistence to shear favours the alignment of the representative cell in the parallel and perpendicular orientations. Instead, in the first row of Fig. 5 it can be for instance observed that the regions corresponding to admissible parallel asymptotic orientation (i.e., the cyan and green ones) get larger decreasing $\hat{k}_b$, that is the ratio between the elastic moduli of the two elements *a* and *b*. This suggests that the presence of fibers not all aligned in the same direction favours oblique asymptotic orientations. The fact that softer substrates, i.e., with lower $\hat{k}_s$, are characterised by larger regions with parallel and perpendicular orientations (see the third row in Fig. 5) has been already discussed above.

## Effect of Cell Elongation

**Fig. 6:**
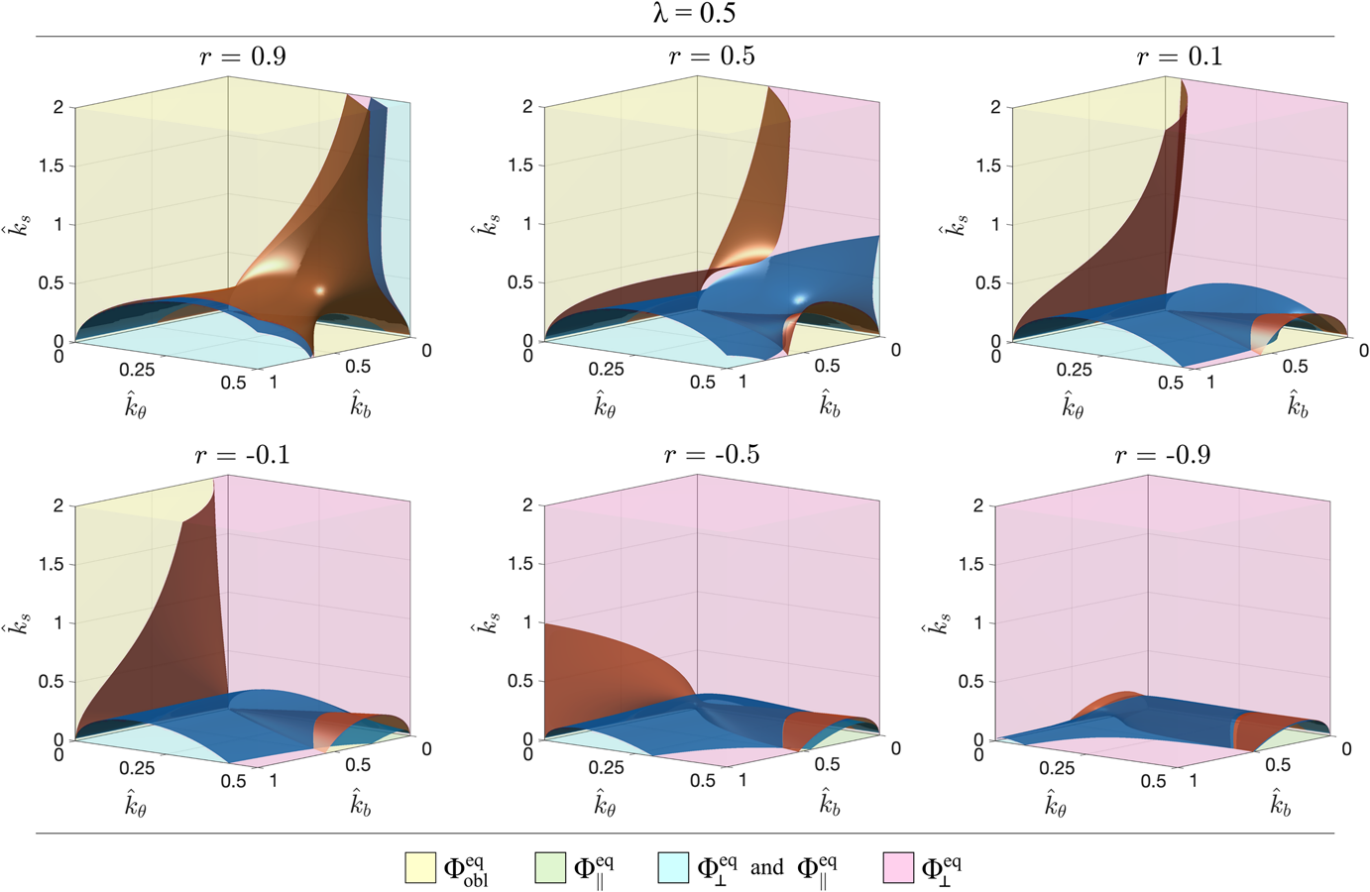
Surfaces $\gamma (\hat{k}_b , \hat{k}_\theta ,\hat{k}_s ,r)=0$ in blue, and to $\beta (\hat{k}_b , \hat{k}_\theta ,\hat{k}_s ,r)=0$ in brown that delimit the different scenarios in the case of elongated cells with $\lambda =0.5$, for different values of *r*, positive in the top row, negative below

**Fig. 7:**
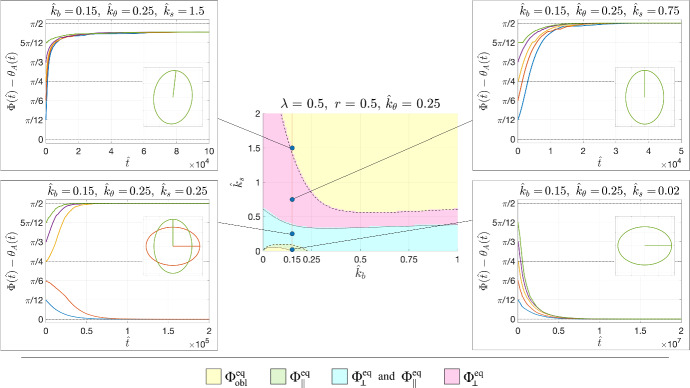
Examples of evolutions of the orientations of an elongated cell in response of an imposed strain ${\varepsilon }(\hat{t})=0.05\left( 1-\cos \frac{\hat{t}}{10}\right)$; toward $\Phi _{obl }^{eq }$ in the yellow region (top left); $\Phi _\perp ^{eq }$ in the magenta region (top right); either $\Phi _\Vert ^{eq }$ or $\Phi _\perp ^{eq }$ according to the initial condition in the cyan region (bottom left); only $\Phi _\Vert ^{eq }$ in the green region (bottom right). The dotted line corresponds to $\beta =0$ and the full line to $\gamma =0$. In the insets a sketch of the final oriented cell shape(s) is reported

**Fig. 8:**
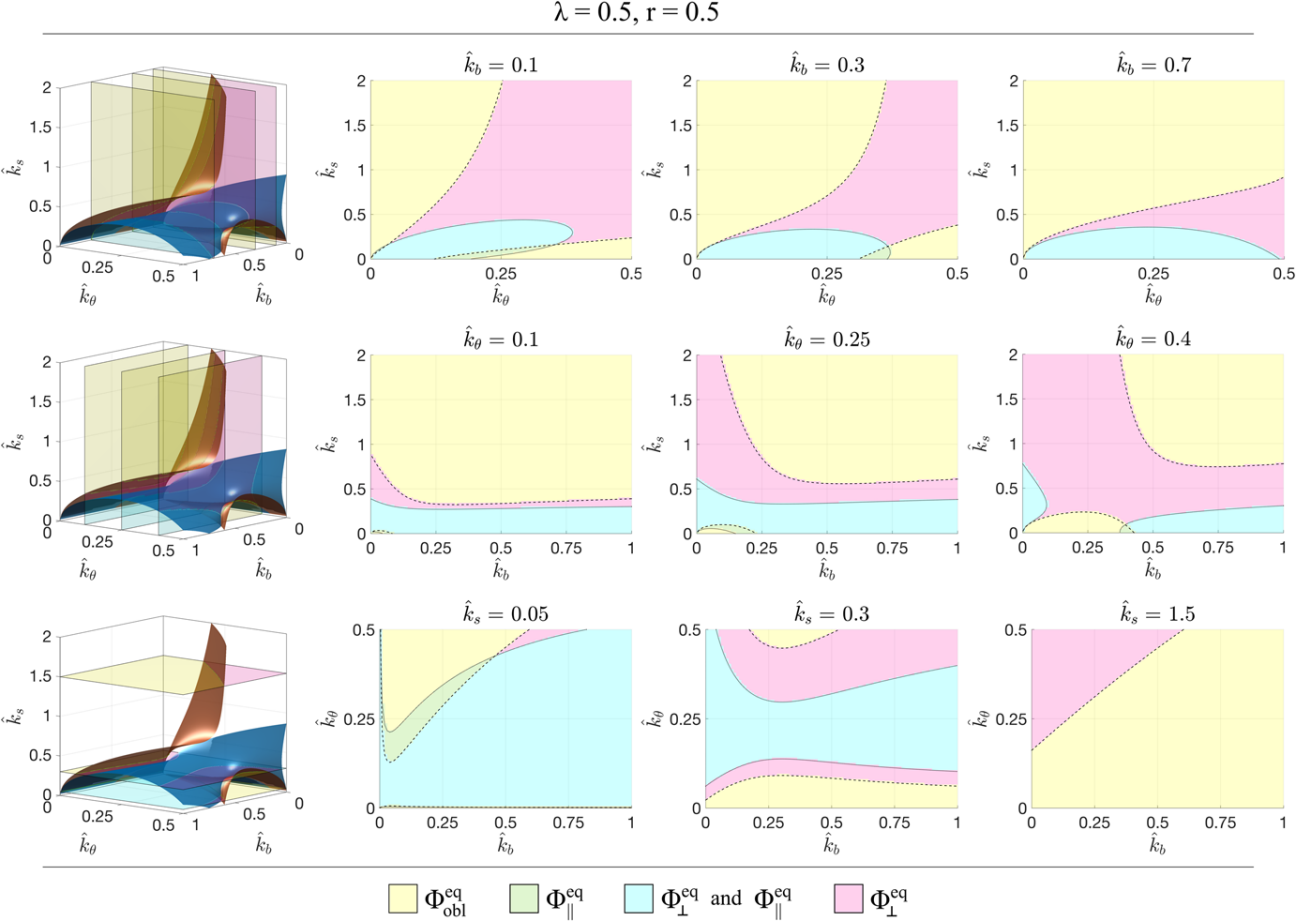
Sections for constant $\hat{k}_b$ (top row), constant $\hat{k}_\theta$ (central row), and constant $\hat{k}_s$ (bottom row) for $\lambda =1$ and $r=0.5$. The dotted line corresponds to $\beta =0$ and the full line to $\gamma =0$ (Color figure online)

In this section we will focus on the effect of the aspect ratio $\lambda$ of the representative cell on its asymptotic behaviour. Generally speaking, referring to Fig. 6 it can be observed that the cyan region characterised by the trend either to $\Phi _\Vert ^{eq }$ or $\Phi _\perp ^{eq }$ (with $\Phi _{obl }^{eq }$ unstable) becomes much smaller. In particular, the magenta region where the asymptotic trend of cell orientation is toward $\Phi _\perp ^{eq }$ enlarges considerably. The yellow region related to $\Phi _{obl }^{eq }$ slightly changes w.r.t. to the case with $\lambda =1$ when *r* is larger than 0.5. Conversely, for smaller values of *r*, the yellow region decreases at the benefit of magenta region related to $\Phi _\perp ^{eq }$. This is even more evident for negative values of *r*. In fact, the region even disappears as *r* gets closer to $-1$. Further details on the evolution and on the regions are given in Figs. 7 and 8.

This means that, on soft substrates, round-like cells more likely orient themselves in a parallel or perpendicular fashion with respect to the main stretching directions, than elongated cells, that prefer the perpendicular orientation also with respect to the oblique one. So, cells like fibroblasts and smooth muscle cells, that are typically characterised by a large aspect ratio, are more prone to keep an oblique or perpendicular orientation even on soft substrates with respect to endothelial or epithelial cells, that usually keep an almost round shape, even under stretch.

## Conclusions

We have modelled the behaviour of cells seeded on an elastic substrate undergoing a periodic deformation using a discrete model for the cell interacting with the continuum substrate, focusing on the effect of the mechanical properties of the cells and above all of the substrate. Specifically, we schematised the cell as formed by two elastic elements, modelling its stress fiber/focal adhesion complexes along its main and transversal orientations, hinged at the center through a torsional spring.

The main focus of the present work was to describe the possible asymptotic scenarios as a function of the mechanical characteristics of the substrate. Consistently with the experiments, it is found that the supercritical scenario characterised by the presence of a stable oblique configuration is favoured by sufficiently large substrate stiffness, while the subcritical scenario characterised by the stability of parallel and perpendicular orientations is favoured by softer substrates. In addition, the region characterised by subcriticality (i) increases as $\hat{k}_b$ (measuring the ratio of the cell response along the direction perpendicular to the main cell orientation axis versus the latter) decreases and (ii) increases as $\hat{k}_\theta$ (measuring the ratio of the cell response to shear versus the one along the main cell orientation axis) increases.

Finally, we studied the effect of cell elongation, finding that the region of the mechanical parameters characterised by a subcritical scenario decreases with cell elongation. This means that oblique orientations with respect to the main stretching direction are more easily mantained by elongated cells such as fibroblasts and smooth muscle cells, than by rounder cells such as epithelial or endothelial cells.

We also showed that the r.h.s. of the ordinary differential Eq. (9) governing cell reorientation presents a factor $\dfrac{\hat{k}_s ^2}{(1+\hat{k}_s )^2}$ that leads to a considerable increase of the reorganization time as the substrate softens. Specifically, this means that if the stiffness decreases by one order of magnitude (e.g., from about 1MPa for silicon elastomers and polydimetylsiloxane (PDMS), to about 100 KPa of collagen gels, or even less), we can expect the reorientation time to increase by two orders of magnitude (i.e., from a couple of hours to more than a week). This practically implies that over very soft substrates reorientation is not observed in the time of the experiment and is easily hidden by random behaviours, as explained in Loy and Preziosi (in press).

As the discrete cell model is based on elastic-like elements, it is not able to describe the dependence of cell behaviour on the frequency of the imposed deformation, i.e., the experimental fact that, roughly speaking, oscillations need to be fast enough (say, higher than 0.1 Hz) to trigger cell reorientation. In order to do that, following what done in Giverso et al. (2021), one should include viscoelastic effects relative to the remodelling of focal ahesions and to the viscoelastic behaviour of the cytoskeleton. This might also take into account of the behaviour of slip bonds and of the development of catch bonds in response to the imposed traction. However, the modelling approach proposed here can be properly generalised to include such effects by adding viscous elements to the struts and to the torsional spring, possibly time dependent and stress dependent, so that they become Maxwell-like elements. Actually, with respect to Giverso et al. (2021), the modelling structure proposed here has the advantage to allow to discriminate between the reaction of the cytoskeleton from the one due to focal adhesions reorganization, here treated as a single structure. In a similar way, it would be interesting to include the active response of the acto-myosin machinery to the imposed deformation, once this effect is clarified in experiment.

## Appendix A

Calculation and minimization of the total energy stored by the system as a consequence of the imposed substrate deformation.

Given the substrate deformation gradient $\mathbb {F}$ in Eq. (1), we have that

17 $$\begin{aligned} \textbf{x}_{S _A }= & {} \mathbb {F}{\textbf {X}}_{S _A }=(1+{\varepsilon })L_a \cos \Phi \,\textbf{i}+(1-r{\varepsilon })L_a \sin \Phi \,\textbf{j}, \end{aligned}$$

18 $$\begin{aligned} \textbf{x}_{S _B }= & {} \mathbb {F}{\textbf {X}}_{S _B }=-(1+{\varepsilon })L_b \sin \Phi \,\textbf{i}+(1-r{\varepsilon })L_b \cos \Phi \,\textbf{j}. \end{aligned}$$

Equations (17)–(18) are obtained recalling that, before the imposed external stress,

19 $$\begin{aligned} {} {\textbf {X}}_{S _A }= & {} {\textbf {X}}_A =L_a \,(\cos \Phi \,\textbf{i}+\sin \Phi \,\textbf{j}), \end{aligned}$$

20 $$\begin{aligned} {} {\textbf {X}}_{S _B }= & {} {\textbf {X}}_B =-L_b \,(\sin \Phi \,\textbf{i}+\cos \Phi \,\textbf{j}). \end{aligned}$$

Due to the elastic characteristics of the cell cytoskeleton, the end points of the elements instead go to

21 $$\begin{aligned} \textbf{x}_A= & {} l_a \cos (\Phi -\theta _a )\,\textbf{i}+l_a \sin (\Phi -\theta _a )\textbf{j}, \end{aligned}$$

22 $$\begin{aligned} \textbf{x}_B= & {} -l_b \sin (\Phi +\theta _b )\,\textbf{i}+l_b \cos (\Phi +\theta _b )\textbf{j}. \end{aligned}$$

In the limit of small deformations, Eqs. (21)–(22) simplify to

23 $$\begin{aligned} \textbf{x}_A\approx & {} [L_a \cos \Phi +\xi _a \cos \Phi +L_a \sin \Phi \,\theta _a ]\,\textbf{i}+[L_a \sin \Phi +\xi _a \sin \Phi -L_a \cos \Phi \,\theta _a ]\,\textbf{j},\nonumber \\ \end{aligned}$$

24 $$\begin{aligned} \textbf{x}_B\approx & {} [-(L_b +\xi _b )\sin \Phi -L_b \cos \Phi \,\theta _b ]\,\textbf{i}+[(L_b +\xi _b )\cos \Phi -L_b \sin \Phi \,\theta _b ]\,\textbf{j},\nonumber \\ \end{aligned}$$

being $l_a = L_a +\xi _a$ and $l_b = L_b +\xi _b$. From Eqs. (21)–(22) and Eqs. (23)–(24), we can then calculate

25 $$\begin{aligned} \textbf{x}_{S _A }-\textbf{x}_A\approx & {} [({\varepsilon }L_a -\xi _a )\cos \Phi -L_a \sin \Phi \,\theta _a ]\,\textbf{i}+[-(r{\varepsilon }L_a +\xi _a )\sin \Phi +L_a \cos \Phi \,\theta _a ]\,\textbf{j},\nonumber \\ \end{aligned}$$

26 $$\begin{aligned} \textbf{x}_{S _B }-\textbf{x}_B\approx & {} [-({\varepsilon }L_b -\xi _b )\sin \Phi +L_b \cos \Phi \,\theta _b ]\,\textbf{i}+[-(r{\varepsilon }L_b +\xi _b )\cos \Phi +L_b \sin \Phi \,\theta _b ]\,\textbf{j},\nonumber \\ \end{aligned}$$

so that

27 $$\begin{aligned} |\textbf{x}_{S _A }-\textbf{x}_A |^2\approx & {} {\varepsilon }^2 L_a ^2(\cos ^2\Phi +r^2\sin ^2\Phi ) - 2{\varepsilon }L_a ^2(1+r)\sin \Phi \cos \Phi \,\theta _a + L^2_a \theta _a ^2 + \nonumber \\{} & {} -\,2{\varepsilon }L_a \xi _a (\cos ^2\Phi -r\sin ^2\Phi ) + \xi _a ^2, \end{aligned}$$

28 $$\begin{aligned} |\textbf{x}_{S _B }-\textbf{x}_B |^2\approx & {} {\varepsilon }^2 L_b ^2(\sin ^2\Phi +r^2\cos ^2\Phi ) - 2{\varepsilon }L_b ^2(1+r)\sin \Phi \cos \Phi \,\theta _b +L^2_b \theta _b ^2 \nonumber \\{} & {} + 2{\varepsilon }L_b \xi _b (r\cos ^2\Phi -\sin ^2\Phi ) +\xi _b ^2. \end{aligned}$$

Putting Eqs. (27)–(28) in Eq. (2), we finally obtain Eq. (3).

The equilibrium configuration of the cell achieved in such a first phase of the process can be then identified by minimizing $U_{\text {tot}}$, i.e., by solving the following system:

29 $$\begin{aligned} \left\{ \begin{array}{l} \dfrac{\partial U_{\text {tot}}}{\partial \xi _a }=-k_a \xi _a -k_s [\xi _a -{\varepsilon }L_a (\cos ^2\Phi -r\sin ^2\Phi )]=0,\\ \dfrac{\partial U_{\text {tot}}}{\partial \xi _b }=-k_b \xi _b -k_s [\xi _b +{\varepsilon }L_b (r\cos ^2\Phi -\sin ^2\Phi )]=0,\\ \dfrac{\partial U_{\text {tot}}}{\partial \theta _a }=-k_\theta (\theta _a +\theta _b )-k_s L_a ^2[\theta _a -{\varepsilon }(1+r)\sin \Phi \cos \Phi ]=0,\\ \dfrac{\partial U_{\text {tot}}}{\partial \theta _b }=-k_\theta (\theta _a +\theta _b )-k_s L_b ^2[\theta _b -{\varepsilon }(1+r)\sin \Phi \cos \Phi ]=0,\\ \end{array} \right. \end{aligned}$$

which implies that

30 $$\begin{aligned} \left\{ \begin{array}{l} \bar{\xi }_a =\dfrac{{\varepsilon }L_a k_s }{k_a +k_s } (\cos ^2\Phi -r\sin ^2\Phi ),\\ \bar{\xi }_b =-\,\dfrac{{\varepsilon }L_a k_s }{k_b +k_s } (r\cos ^2\Phi -\sin ^2\Phi ),\\ \bar{\theta }_ab :=\bar{\theta }_a + \bar{\theta }_b =\dfrac{2{\varepsilon }k_s }{\left( \frac{1}{L_a ^2}+\frac{1}{L_b ^2}\right) k_\theta +k_s }(1+r)\sin \Phi \cos \Phi . \end{array}\right. \end{aligned}$$
